# Novel MRI metrics: Is targeting brain atrophy a practical treatment goal? No

**DOI:** 10.1177/13524585261433897

**Published:** 2026-03-29

**Authors:** Eva M.M. Strijbis, Menno M. Schoonheim

**Affiliations:** Department of Neurology, Amsterdam UMC, Amsterdam, The Netherlands; Department of Anatomy and Neurosciences, Clinical Neuroscience, MS Center Amsterdam, Amsterdam UMC, Amsterdam, The Netherlands

Preventing slow disability progression remains one of the most important unmet needs in MS. It is known to be especially impacted by neurodegeneration in the form of irreversible neuro-axonal damage, which can be captured on magnetic resonance imaging (MRI) as brain atrophy.^1^ On a group level, brain atrophy is associated with cognitive and physical disability, and progression independent of relapse activity—more so than lesion burden.^2^

But while lesion detection on MRI remains the foundation of diagnosing MS, the use of volume measurements for prognostication and as a treatment goal is still not implemented in clinics for individual patients.^3^ While this is not due to a lack of conceptual relevance or expert agreement,^4^ unresolved methodological and practical issues currently prevent the use of brain atrophy measurements as a practical treatment goal.

It has been more than 10 years since the first controversy on this topic,^5^ and unfortunately, most arguments against its use for treatment monitoring remain. One issue lies in the sensitivity of the measure—that is, the ability to detect subtle changes in brain volume beyond inherent noise, especially when using clinical MRI protocols. Yearly atrophy rates in MS have been shown to be about double those of healthy controls, yet they remain relatively small (0.5%–1%).^1^ The detection of such small changes in individual patients is difficult with the current resolution of MRI protocols, as well as due to inherent noise and variability in repeated MRI measurements. Variability in scan–rescan measurements has improved, yet still overlaps with a significant portion of disease-specific change.^6^ This noise increases significantly when different protocols and MRI machines are used. In fact, estimates of measurement error suggest that brain atrophy in an MS patient can be reliably observed over 1–2 years under the most homogeneous conditions (e.g. using the same scanner). It may require up to 5–10 years for reliable detection when different scanners are used,^7^ and it may come as no surprise that requiring the same scanner for individual patients over a period of years is often not feasible in a routine (non-academic) clinical setting.

Apart from technical challenges, there are also physiological considerations that have been previously published.^4^ In contrast to the development and resolution of focal inflammation, brain atrophy in MS is a slow process. Brain volume loss only becomes apparent after 1- to 2-year windows, making longitudinal volumetric change a difficult target for practical application. In addition, brain atrophy is a normal part of aging, which is difficult to disentangle from MS-driven neurodegeneration. Some even consider atrophy in MS to be a form of accelerated aging and increasing “brain age.”^8^ This concept has also yielded novel ways of quantifying the severity of neurodegeneration compared to what one would expect in a normal aging sample, which might open doors to more sensitive measures—for example, using deep learning approaches—after additional validation steps.

In addition, MS is not the only factor that can accelerate brain atrophy. Lifestyle factors such as smoking, diabetes, and cardiovascular comorbidities negatively affect brain volumes as well. Normal variations in anatomy, sex differences, body size and height, hydration, and even hormonal status also impact volumetric measurements.^4^ To correct for some of these variations, techniques have been developed that use a person’s skull or intracranial volume or that convert measurements to Z-scores based on a comparative healthy sample. For longitudinal evaluations, such corrections may be easier, as a person’s brain volume at baseline can serve as their own control. What is direly needed, however, is a normative dataset to determine at what point brain volume loss becomes a significant concern—that is, a specific threshold when consideration of treatment escalation becomes relevant. Such normative data are currently being developed, both for normal aging and MS progression, with the aforementioned technical difficulties as an important caveat.^9^

The aim of ultimately using brain atrophy to influence treatment strategies also warrants caution regarding the presence of “pseudo-atrophy.”^4^ This refers to the phenomenon of accelerated brain shrinkage after treatment initiation due to the resolution of lingering edema surrounding areas of acute inflammation. More severe inflammation can therefore mask the true neurodegenerative status. To manage this period, during which accurate quantification of atrophy is difficult, so-called re-baselining is recommended—waiting 6–12 months after treatment escalation before evaluating neurodegenerative changes.

The balance between accurate measurement and clinical relevance also remains an important topic. Whole-brain volumes are the most accurate to quantify, but their relationship with clinical performance is weak. Zooming in on individual structures like the thalamus and spinal cord yields stronger clinical correlations, but these measurements are noisier. Thalamic atrophy occurs early in the disease course, sometimes even before a definitive MS diagnosis, and it has been shown to correlate more strongly with clinical progression than whole brain atrophy.^10^ However, given the relatively low contrast between thalamic and white matter tissue, accurate segmentation typically requires complex software packages, which might not be feasible in non-academic clinical settings. In addition, the required 3D T1 images are not always part of the clinical scanning protocol. Spinal cord volume also has clear and strong clinical correlations and can now be assessed using cervical spine images without dedicated spinal cord imaging, although similar software limitations apply. Consensus recommendations on the use of atrophy in MS have now been published,^4^ hopefully leading to concrete cut-offs and guidelines for how neurologists should use this information in daily practice.

Taken together, it is clear that quantifying neurodegeneration in MS remains an important unmet need, and that strategies to bring these measures into clinics are still evolving. They, however, still require validation of MS-specific models to determine clinical cut-off scores and guidelines, adjustment of clinical protocols or validation of fluid-attenuated inversion recovery (FLAIR)-based measurements (given the frequent absence of 3D T1 imaging in hospital settings) and further refinement of artificial intelligence (AI)-based methods to reduce noise in longitudinal measurements and for automated segmentations that are low-cost in terms of processing time and standardized reporting. As such, while currently targeting brain atrophy in MS is not yet a practical treatment goal, it hopefully—finally—will be in the coming decade.

## References

[1] De Stefano N Stromillo ML Giorgio A , et al. Establishing pathological cut-offs of brain atrophy rates in multiple sclerosis. J Neurol Neurosurg Psychiatry 2016; 87(1): 93–99. 25904813 10.1136/jnnp-2014-309903 PMC4717444

[2] Cagol A Schaedelin S Barakovic M , et al. Association of brain atrophy with disease progression independent of relapse activity in patients with relapsing multiple sclerosis. JAMA Neurol 2022; 79(7): 682–692. 35575778 10.1001/jamaneurol.2022.1025 PMC9112138

[3] Van Nederpelt DR Mendelsohn ZC Bos L , et al. User requirements for quantitative radiological reports in multiple sclerosis. Eur Radiol 2025; 35(10): 5967–5978. 40240557 10.1007/s00330-025-11544-x PMC12417231

[4] Sastre-Garriga J Pareto D Battaglini M , et al. MAGNIMS consensus recommendations on the use of brain and spinal cord atrophy measures in clinical practice. Nat Rev Neurol 2020; 16(3): 171–182. 32094485 10.1038/s41582-020-0314-x PMC7054210

[5] Filippi M Rocca MA. Preventing brain atrophy should be the gold standard of effective therapy in MS (after the first year of treatment): No. Mult Scler 2013; 19(8): 1005–1006. 23818019 10.1177/1352458513482387

[6] Van Nederpelt DR Amiri H Brouwer I , et al. Reliability of brain atrophy measurements in multiple sclerosis using MRI: An assessment of six freely available software packages for cross-sectional analyses. Neuroradiology 2023; 65(10): 1459–1472. 37526657 10.1007/s00234-023-03189-8 PMC10497452

[7] Biberacher V Schmidt P Keshavan A , et al. Intra- and interscanner variability of magnetic resonance imaging based volumetry in multiple sclerosis. Neuroimage 2016; 142: 188–197. 27431758 10.1016/j.neuroimage.2016.07.035

[8] Skattebøl L Nygaard GO Leonardsen EH , et al. Brain age in multiple sclerosis: A study with deep learning and traditional machine learning. Brain Commun 2025; 7(3): fcaf152. 10.1093/braincomms/fcaf152 PMC12056726 40337466

[9] Van Nederpelt DR Bos L Mattiesing RM , et al. Multiple sclerosis-specific reference curves for brain volumes to explain disease severity. Neurology 2025; 104(10): e213618. 10.1212/WNL.0000000000213618 PMC12012623 40267375

[10] Eshaghi A Prados F Brownlee WJ , et al. Deep gray matter volume loss drives disability worsening in multiple sclerosis. Ann Neurol 2018; 83(2): 210–222. 29331092 10.1002/ana.25145 PMC5838522

